# The wider expansion of pediatric robotic urology: exploring the patterns of adoption in complex bladder reconstruction

**DOI:** 10.1007/s11701-026-03903-7

**Published:** 2026-09-22

**Authors:** Mehmet Erol Maras, Zeeshan Zubair, Priyank Yadav, Huzeyfe Yusuf Sahin, Kareem Touleimat, Marwan Hamadeh, Berk Burgu, Mohan Gundeti

**Affiliations:** 1 Department of Urology, Ankara University Faculty of Medicine, Ankara, 06230 Turkey; 2 Department of Urology, University of Illinois College of Medicine, Chicago, IL USA; 3 Department of Urology, Rosalind Franklin University of Medicine and Science, North Chicago, IL USA; 4 Department of Pediatric Urology, Ankara University Faculty of Medicine, Ankara, Turkey; 5 Section of Urology, Department of Surgery, The University of Chicago Medical Center, 5841 S. Maryland Avenue, MC 6038, Chicago, 60637 IL USA

**Keywords:** pediatric robotic surgery, lower urinary tract reconstruction, appendicovesicostomy, augmentation cystoplasty, bladder neck reconstruction

## Abstract

Robotic surgery is increasingly used in pediatric urology, particularly for moderate-complexity procedures such as pyeloplasty and ureteral reimplantation. Whether this expansion has extended to high-complexity lower urinary tract (LUT) reconstruction remains unclear. To characterize global trends in robot-assisted appendicovesicostomy (APV), augmentation cystoplasty, and bladder neck reconstruction (BNR) between March 2016 and March 2025, and to evaluate changes in proportional robotic adoption. A systematic review of PubMed, Embase, and Scopus (PROSPERO CRD420251105224) identified English-language studies reporting robotic APV, augmentation cystoplasty, or BNR in patients ≤18 years. Eligible studies included case reports, case series, and comparative studies. Two reviewers independently extracted study characteristics, operative outcomes, and annual case volumes. Published epidemiologic data were used to estimate yearly global robotic adoption rates. Thirty-six publications comprising 329 robotic procedures were included: 192 APV (58%), 70 augmentation cystoplasties (21%), and 67 BNRs (20%). Annual case volumes fluctuated without a sustained upward trend. APV accounted for most reported procedures, with publication-year peaks in 2017, 2019, and 2025. Although overall pediatric robotic urologic volume increased nearly threefold between the historical and contemporary eras (3,688 vs. 10,907 cases), estimated robotic adoption for APV, augmentation cystoplasty, and BNR remained below 10% for every procedure across all years. Functional outcomes and complication rates were generally comparable to historical open series. Contemporary growth in pediatric robotic urology has been driven primarily by lower-complexity procedures, while adoption for complex LUT reconstruction remains limited. Broader implementation will require advances in technology, structured training, and resource allocation.

## Introduction

Robotic technology has transformed the field of pediatric urology over the past two decades. In 2002, the first pediatric urologic robotic procedure performed was the robotic-assisted laparoscopic pyeloplasty [1]. Shortly thereafter, additional reconstructive applications emerged, including robotic ureteral reimplantation, first described in 2004 [2]. Owing to their relatively high procedural volume and lower technical complexity compared with more advanced reconstructions, these operations rapidly became the standard entry points for pediatric robotic surgery and now constitute the majority of pediatric robotic cases reported worldwide.

As familiarity with the robotic interface increased, more complex procedures were progressively introduced. By the late 2000s, pioneering groups described robotic approaches to appendicovesicostomy (APV), augmentation cystoplasty, and bladder neck reconstruction (BNR) – among the most technically demanding forms of pediatric lower urinary tract (LUT) reconstruction, requiring meticulous dissection and intricate intracorporeal suturing [3–5]. In parallel with the global expansion of robotic platforms, attempts were made to adapt the technology for smaller patients, including the introduction of 5-mm ports and instruments in addition to standard 8-mm ports. Although these smaller instruments were subsequently discontinued in newer generation robots, contemporary systems retained the dexterity and precision necessary for complex pediatric reconstructions, while newer console and arm designs have reduced the working space required in the operating room. Provided that port spacing of at least 6-cm can be achieved, these platforms can be safely deployed in the constrained abdominal cavities of children.

Despite these technical advances, it remains unclear whether robotic platforms have achieved meaningful and sustained integration into high-complexity pediatric reconstructive practice. Prior bibliometric work – most notably the analysis by Cundy et al. – catalogued global trends in pediatric robotic surgery as a whole, focusing predominantly on pyeloplasty and ureteral reimplantation [6]. However, the study primarily reflected an earlier era of adoption and did not specifically evaluate whether increasing robotic case volume has been accompanied by a shift toward higher procedural complexity or by a broader uptake of robotic techniques for complex bladder and continence procedures.

The present study seeks to address this gap by analyzing global publication trends between 2016 and 2025 for robotic APV, augmentation cystoplasty, and BNR in children. We selected these procedures because of their technical complexity, traditional predominance as open operations and relatively low procedural volume in pediatric practice [7]. Our primary objective was to determine whether increased availability and utilization of robotic platforms in the contemporary era have translated into greater proportional adoption of robotic techniques for high-complexity LUT reconstructions. To contextualize contemporary trends, we contrasted our findings with previously reported data from 2002 to 2016. We hypothesized that, despite growth in overall pediatric robotic case volume, relative adoption of robotics for complex LUT reconstruction remains low and unevenly distributed.

## Methods

### Study design and setting

This study was a descriptive systematic review designed to analyze global publication and adoption trends in pediatric robotic LUT reconstruction. We focused on three index procedures–APV, augmentation cystoplasty and BNR–performed in patients aged ≤ 18 years between March 2016 and March 2025. The review protocol was registered with the International Prospective Register of Systematic Reviews (PROSPERO; registration number CRD420251105224) and conducted in accordance with the Preferred Reporting Items for Systematic Reviews and Meta-Analyses (PRISMA) 2020 guidelines.

To contextualize contemporary trends, we contrasted the 2016–2025 period with an earlier era of pediatric robotic urologic surgery described by Cundy et al. [6] For temporal trend comparison, the contemporary study period (2016–2025) was contrasted with the previously published era (2002–2016), allowing evaluation of changes in robotic adoption over two sequential time intervals.

## Eligibility criteria

We included peer-reviewed original research articles, case series, case reports, and conference abstracts that met the following criteria:

- Population: pediatric patients aged ≤ 18 years.
- Intervention: robot-assisted (or robotic-assisted laparoscopic) APV, augmentation cystoplasty, or BNR.
- Outcomes: explicit reporting of the number of robotic procedures performed, with discrete counts for at least one of the three index procedures.
- Language: English.
- Time frame: March 31, 2016, to March 31, 2025 (publication date).

Studies were excluded if:

- They combined adult and pediatric populations without separate pediatric stratification.
- They did not specify the type or number of robotic procedures.
- They reported purely experimental, simulation, or cadaveric work without clinical cases.

Studies reporting more than one index procedure were categorized under each relevant procedural group. Consequently, procedure counts represent operations rather than unique patients, and patients undergoing combined reconstructions may be represented in more than one procedural category.

## Data sources and search strategy

A comprehensive literature search was conducted using PubMed, Embase, and Scopus. The search strategy combined controlled vocabulary terms and free-text keywords related to robotic surgery, pediatric urology, and the index procedures. Core terms included combinations of:

- “robotic” OR “robot-assisted”.
- “pediatric” OR “child” OR “children”.
- “appendicovesicostomy” OR “Mitrofanoff”.
- “bladder augmentation” OR “augmentation cystoplasty”.
- “bladder neck reconstruction” OR “bladder neck surgery”.

Database-specific subject headings (e.g., MeSH, Emtree) were incorporated where appropriate, and search strings were adapted to each platform’s syntax.

**Fig. 1:**
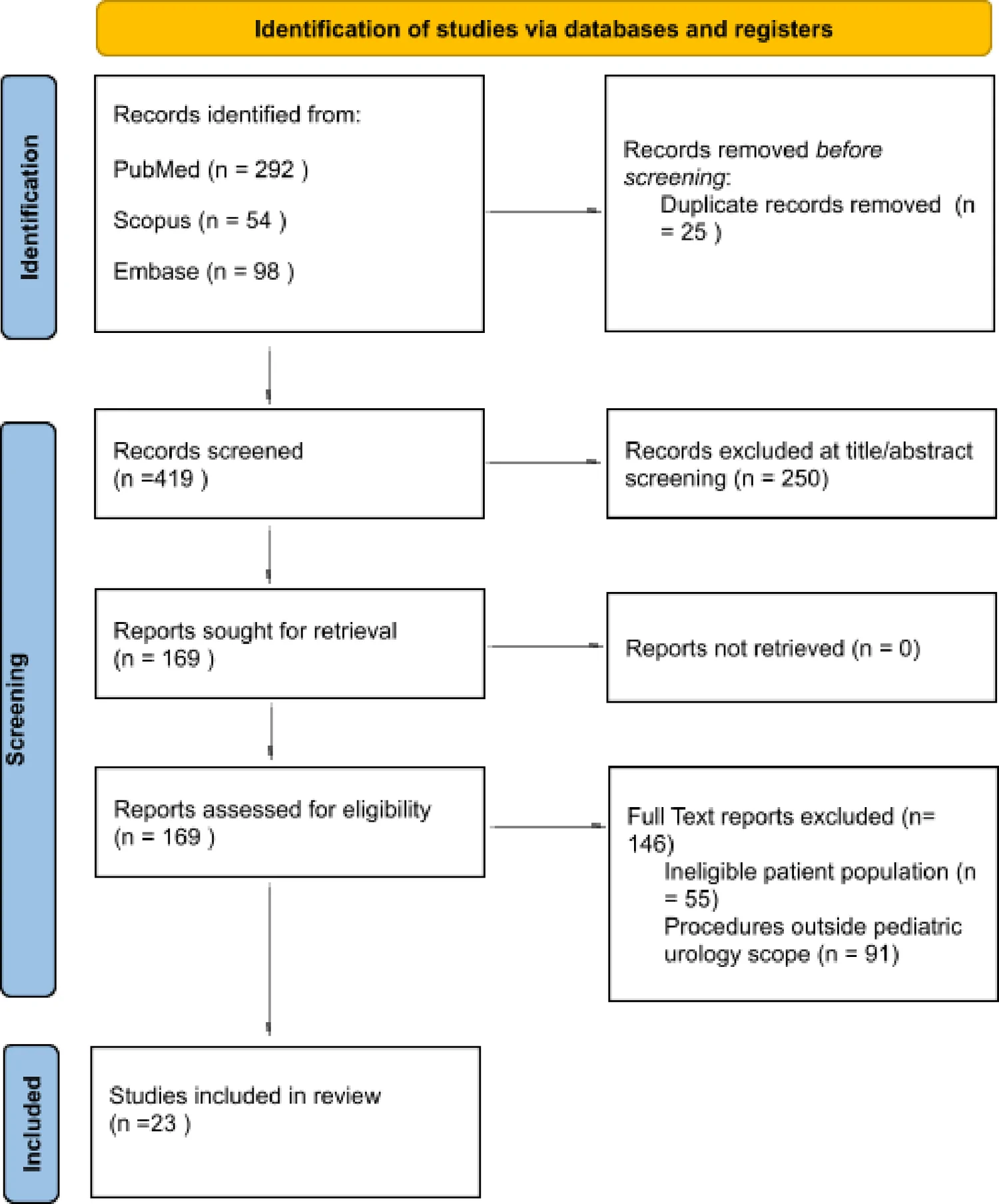
PRISMA flow diagram showing study methodology

After deduplication, two reviewers (M.E.M. and H.Y.S.) independently screened titles and abstracts to identify potential eligible studies. Full-texts were obtained and reviewed for all records that met inclusion criteria or where eligibility remained uncertain. Disagreements were resolved through discussion or adjudication by a senior author. The study selection process is detailed in the PRISMA 2020 flow diagram (Fig. 1).

## Data extraction

Two reviewers independently extracted data using a standardized data collection form. Extracted variables included:

- **Study-level characteristics**: first author, year of publication, country/region, study setting (single-institution vs. multi-institutional), study design (case report, case series, comparative study), and time frame of case performance (e.g., 2014–2019).
- **Procedure-level data**: number of robotic APV, augmentation cystoplasty, and BNR procedures; whether multiple procedures were performed in the same patient (e.g., augmentation + APV).
- **Patient-level characteristics (when available)**: age, sex, underlying diagnosis (e.g., neurogenic bladder, posterior urethral valves, tethered cord syndrome, prune belly syndrome), and indication for surgery.
- **Operative and perioperative outcomes (when available)**: operative time, estimated blood loss, length of hospital stay, continence outcomes, reintervention rates, follow-up duration, and postoperative complications (graded by Clavien–Dindo classification where reported).

## Estimation of global procedural volumes and adoption rates

To estimate proportional adoption of robotic techniques, we derived annual denominators for total APV, augmentation cystoplasty, and BNR volumes and for overall pediatric robotic urologic surgery.

- **Historical era (2002–2016)**: For the earlier era, we used data from the bibliometric analysis by Cundy et al. [6] and primary references cited therein to identify reported pediatric robotic APV, augmentation, and BNR cases between 2002 and 2016. Using the same methodology as our contemporary review, we identified approximately 286 robotic complex LUT reconstruction procedures over this 14-year interval (APV, augmentation cystoplasty, and BNR combined). Previously published estimates of total pediatric robotic urologic case volume from Cundy et al. were used to derive an approximate annual average of 250 pediatric robotic procedures during this period.
- **Contemporary era (2016–2025) – total pediatric robotic urologic volumes**: To estimate the contemporary denominator of overall pediatric robotic urologic volume, we performed a parallel search of pediatric robotic urology publications in PubMed, Embase, and Scopus covering March 31, 2016, to March 31, 2025. Two reviewers independently screened and extracted case counts from all eligible publications reporting pediatric robotic urologic procedures of any type (including pyeloplasty, ureteral reimplantation, and other procedures beyond APV, augmentation, and BNR). Case numbers were obtained from abstracts when clearly stated; when necessary, full texts were reviewed to extract or infer case counts. When individual publications reported cases spanning multiple years, total case numbers were apportioned uniformly across the reported time interval to derive approximate annual case counts (e.g., 30 cases over 3 years were allocated as 10 cases per year). This process yielded an estimated total of 10,907 pediatric robotic urologic procedures between 2017 and 2025, corresponding to a mean of approximately 1,211 cases per year.
- **Contemporary era (2016–2025) – complex LUT reconstruction volumes**: Using the same allocation approach, we identified 329 robotic APV, augmentation cystoplasty, and BNR procedures between 2016 and 2025. For each publication, the calendar years during which surgeries were performed were identified (e.g., 2015–2019), and the total number of cases was evenly distributed across those years when year-specific counts were not provided. These allocated annual case counts were then aggregated across all studies to construct estimated annual volumes for each index procedure.
- **Adoption rate calculations**: To characterize proportional adoption of robotics for complex LUT reconstruction, we derived estimated total annual volumes for APV, augmentation cystoplasty, and BNR from previously published epidemiologic reports and large database studies, supplemented by targeted manual extraction from the same databases (PubMed, Embase, Scopus). For each procedure and year, adoption rates were calculated as:
  $$\begin{aligned}\:&{Robotic\:adoption\:rate}_{year,\:procedure}\\&\quad=\frac{{Estimated\:number\:of\:robotic\:cases}_{year,\:procedure}}{{Estimated\:total\:number\:of\:cases}_{year,\:procedure}}\\&\qquad\times\:100\%.\end{aligned}$$
- Because of heterogeneity in reporting and the reliance on published series, these adoption rates were treated as exploratory estimates intended to describe broad patterns rather than precise population-level measures.

## Outcomes and statistical analysis

The primary outcome was the temporal trend in robotic adoption for complex LUT reconstructions, assessed by:

- Annual numbers of robotic APV, augmentation cystoplasty, and BNR procedures.
- Estimated yearly adoption rates (proportion of total cases performed robotically) for each procedure.

Secondary outcomes included:

- Geographic distribution of reported robotic cases.
- Operative time estimated blood loss, length of stay, continence outcomes, reintervention rates, and postoperative complications.

Data were summarized using descriptive statistics. Continuous variables were reported as medians with ranges when available, and categorical variables as frequencies and percentages. No formal hypothesis testing or meta-analysis was performed, given the heterogeneity in study designs, reporting formats, and follow-up durations. All analyses were exploratory and aimed at characterizing patterns of adoption rather than establishing causal relationships.

### Handling of publication year versus operative year

Because many publications reported cases performed over multi-year intervals, we distinguished publication year from operative year in our analyses. Publication-based trends were constructed by assigning all cases to the year of publication. Operative-year trends were constructed by allocating cases to their reported years of performance, as described above. For figures depicting adoption trends, we primarily used operative-year estimates to better reflect the temporal pattern of real-world practice.

## Results

**Fig. 2:**
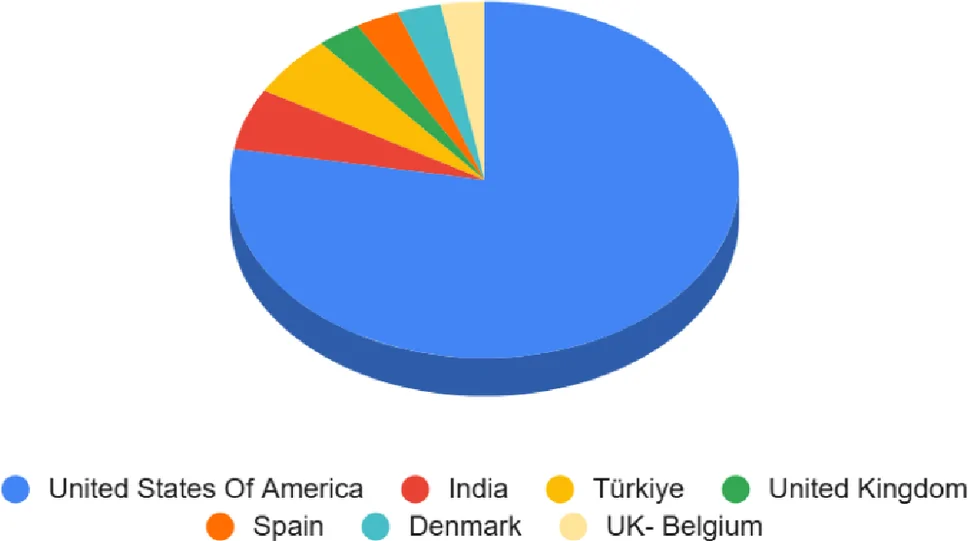
Geographic distribution of robotic cases

A total of 23 publications met the inclusion criteria following systematic screening (Table 1). Among these, 18 reported robotic APV, 9 reported augmentation cystoplasty, and 9 reported BNR. Several studies described more than one index procedure and were therefore represented in multiple procedural categories. Most publications were case series (44.4%) or case reports (44.4%), with comparative studies comprising 11.2%. The United States accounted for the majority of publications (*n* = 28) with additional contributions from India (*n* = 2), Türkiye (*n* = 2), the United Kingdom (*n* = 1), Spain (*n* = 1), Denmark (*n* = 1), and a Belgium–United Kingdom collaborative study (*n* = 1) (Fig. 2). All but one study represented single-institution experiences.

**Table 1:** Study characteristics of included publications

Year	First Author	Last Author	Country/ Region	Study Design	Robotic Cases (n)	Reference
Appendicovesicostomy (APV)						
2017	Huang	Gargollo	USA	Case Report	1	[8]
2017	Fuchs	DaJusta	USA	Case Report	1	[9]
2017	Gargollo	Rocco	USA	Case Series	38	[10]
2017	Ching	DaJusta	USA	Case Report	1	[11]
2018	Halleran	DaJusta	USA	Case Series	6	[12]
2019	Subramaniam	Subramaniam	Belgium–UK	Comparative Study	1	[13]
2019	Adamic	Gundeti	USA	Case Report	1	[14]
2019	Gargollo	DaJusta	USA	Case Series	32	[15]
2019	Subramaniam	Subramaniam	UK	Case Series	18	[16]
2020	Rodriguez	Gundeti	USA	Case Report	1	[17]
2020	Adamic	Gundeti	USA	Case Series	16	[18]
2021	Parikh	Gargollo	USA	Case Report	1	[19]
2021	Galansky	Gundeti	USA	Case Series	34	[20]
2022	Sidelsky	Gundeti	USA	Case Report	1	[21]
2022	Juul	Reinhardt	Denmark	Comparative Study	5	[22]
2023	Bahadır	Ekici	Türkiye	Case Report	1	[23]
2025	Abdulfattah	Shukla	USA	Case Series	33	[24]
2025	Yadav	Chowdhary	India	Case Series	1	[25]
Augmentation Cystoplasty						
2016	Wiestma	N Yu	USA	Case Report	1	[26]
2016	Gundeti	Gargollo	USA	Comparative Study	15	[27]
2017	Kibar	Ergin	Türkiye	Case Series	2	[28]
2019	Adamic	Gundeti	USA	Case Report	1	[14]
2020	Adamic	Gundeti	USA	Case Series	20	[18]
2021	Galansky	Gundeti	USA	Case Series	18	[20]
2022	Sidelsky	Gundeti	USA	Case Report	1	[21]
2024	Rodriguez	Beauregard	Spain	Case Report	1	[40]
2025	Yadav	Chowdhary	India	Case Series	11	[25]
Bladder Neck Reconstruction (BNR)						
2016	Gundeti	Gargollo	USA	Comparative Study	21	[27]
2017	Fuchs	DaJusta	USA	Case Report	1	[9]
2018	Gargollo	Scales	USA	Case Report	1	[29]
2018	Halleran	Gargollo	USA	Case Series	5	[12]
2019	Gargollo	DaJusta	USA	Case Series	27	[15]
2020	Rodriguez	Gundeti	USA	Case Report	1	[17]
2020	Adamic	Gundeti	USA	Case Series	4	[18]
2021	Galansky	Gundeti	USA	Case Series	6	[20]
2022	Sidelsky	Gundeti	USA	Case Report	1	[21]

Studies reporting multiple procedures are represented in more than one procedural category. Procedure counts represent operations rather than unique patients; therefore, patients undergoing combined reconstructions may appear in more than one category

**Fig. 3:**
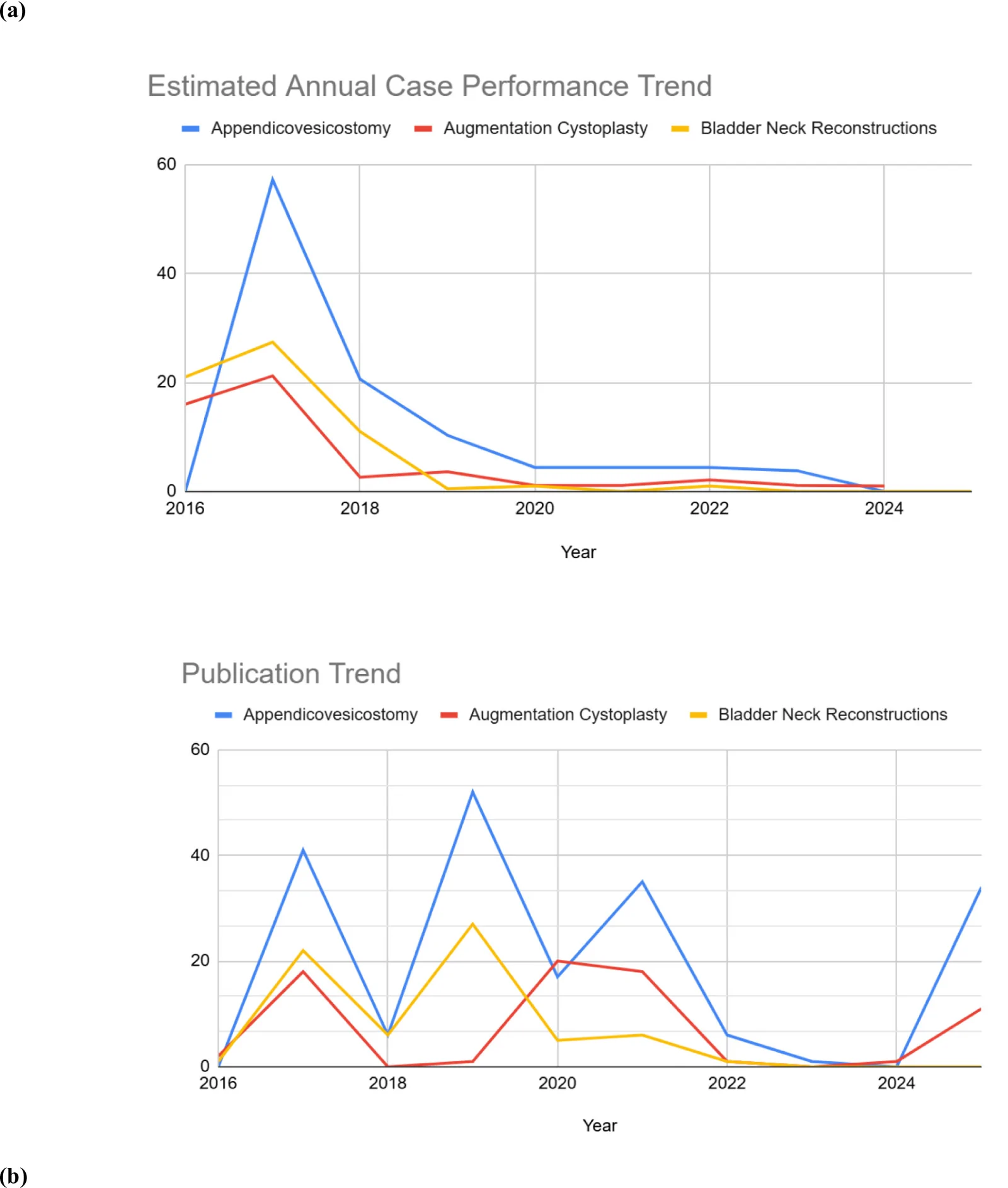
(**a**) Line chart showing estimated annual case performance trend and (**b**) Publication trend

Between March 2016 and March 2025, a total of 329 robotic procedures were reported across the three index reconstructive categories. Of these, 192 cases (58%) were APV, 70 cases (21%) were augmentation cystoplasty, and 67 (21%) were BNR. Annual case volumes fluctuated without a consistent upward trajectory for any procedure type (Fig. 3(a)). When cases were examined by publication year, robotic APV represented the most frequently reported procedure and demonstrated intermittent peaks in 2017 (41 cases), 2019 (52 cases), and 2025 (34 cases). Robotic augmentation cystoplasty reached its highest reported publication-year volume in 2020 (20 cases), while BNR peaked in 2019 (27 cases). No procedure exhibited sustained year-over-year growth in robotic utilization.

To better approximate real-world practice, cases were also reallocated to their years of performance. When examined by estimated operative year, annual volumes of robotic APV, augmentation cystoplasty and BNR remained low and variable throughout the study period, without a clear trend towards increasing adoption (Fig. 3(b)). Across all years, robotic APV rarely exceeded an estimated 8% of total APV volume, and adoption remained even lower for augmentation cystoplasty and BNR, with robotic approaches consistently comprising less than 10% of any index procedure in any given year.

Across all reported procedures, the most common surgical indication was neurogenic bladder secondary to myelodysplasia, followed by posterior urethral valves, tethered cord syndrome, and prune belly syndrome. The overall median patient age at surgery was 10.4 years. Operative details and outcomes are summarized in Table 2. As expected for complex reconstructions, reported operative times were prolonged across all procedures. However, estimated blood loss was consistently low, and lengths of hospital stay were within acceptable ranges for major lower urinary tract reconstruction. Continence outcomes were variably reported but generally favorable among patients with documented follow-up. Reinterventions most commonly involved stomal revision, channel obstruction, or additional bladder neck–related procedures. Complications were predominantly low-grade (Clavien–Dindo I–II), with relatively few high-grade (Clavien–Dindo III–IV) events.

**Table 2:** Patient and operative characteristics by procedure type

Outcome	APV	Augmentation	BNR
Median age (years)	10.3 (5.7–17)	11 (6–17)	10 (6–11.22)
Operative time (min), median	377 (120–615)	454 (332–615)	464 (300–692)
Estimated blood loss (mL), median	50 (5–390)	100 (50–325)	50 (20–103)
Length of stay (days), median	4 (1–27)	7.5 (5.6–28.5)	5.8 (1–14)
Continence achieved (%)	91	100	100
Reintervention (%)	22	17.1	23.7
Clavien–Dindo I–II (%)	33.8	27.3	11.1
Clavien–Dindo III–IV (%)	17.5	18.2	5.6

Outcomes were variably reported across studies. Reintervention was defined as any secondary procedure related to the index reconstruction and reflects patient-level reporting using only studies with explicitly reported data. Complication rates reflect event-based reporting; individual patients may have experienced more than one complication

Historical review of the literature identified 286 reported pediatric APV, augmentation cystoplasty, and BNR procedures performed globally between 2003 and 2016. Using the same methodology, 329 robotic procedures were identified during the 2016–2025 study period, representing an absolute increase in reported complex reconstructive cases. When expressed as annual averages, this corresponds to approximately 20 cases per year in the earlier era versus approximately 37 cases per year in the contemporary period. During the same interval, overall pediatric robotic surgical volume increased nearly threefold (from 3,688 to 10,907 procedures), consistent with an increase in annual average volume from roughly 250 to 1,211 pediatric robotic urologic cases. Despite this expansion, the relative proportion of complex LUT reconstructions performed robotically remained low and largely unchanged over time. Even in the contemporary era, estimated robotic adoption for APV, augmentation cystoplasty and BNR did not exceed 10% for any procedure in any year (Fig. 3).

## Discussion

This systematic review evaluated global trends in robotic APV, augmentation cystoplasty, and BNR in pediatric urology between 2016 and 2025. Despite the widespread availability of robotic platforms and their routine implementation for moderate-complexity procedures such as pyeloplasty and ureteral reimplantation, we found that adoption for high-complexity LUT reconstruction has remained limited. Across nearly a decade of contemporary practice, robotic approaches accounted for fewer than 8% of APV cases annually, with even lower proportional use for augmentation cystoplasty and BNR. No procedure demonstrated a consistent upward trajectory in proportional robotic use, and in no year did the estimated robotic adoption exceed 10% for any index operation. These findings are consistent with and extend prior bibliometric analyses of pediatric robotic surgery. Cundy et al. reported steady growth in overall pediatric robotic urologic volume between 2002 and 2016, driven largely by pyeloplasty and ureteric reimplantation [6]. Our study shows that this global expansion has continued in the contemporary era, with overall pediatric robotic urologic case volume increasing nearly threefold between the earlier and current periods (from 3,688 to 10,907 procedures; annual averages approximately 250 vs. 1,211 cases). In comparison, approximately 286 robotic complex LUT reconstruction procedures (APV, augmentation cystoplasty, and BNR combined) were identified during the historical era (2002–2016), compared with 329 procedures during the contemporary period (March 2016–March 2025). Thus, despite the marked growth in overall pediatric robotic urologic case volume, the absolute increase in reported complex robotic LUT reconstruction has been relatively modest. However, when the adoption is evaluated in a complexity-adjusted, procedure-specific manner, the relative proportion of complex LUT reconstructions performed robotically has been concentrated in moderate-complexity domains rather than reflecting a broad shift towards high-complexity reconstruction. Several factors likely contribute to this stagnation in adoption for complex LUT procedures. From a technical standpoint, augmentation cystoplasty and BNR require advanced intracorporeal suturing, extensive pelvic dissection, and prolonged operative times, making them among the most demanding procedures in pediatric urology [3, 4, 5, 30]. The learning curve for performing these operations is steep, particularly in low-volume pediatric centers where opportunities for repetition are limited [31, 32]. Limited exposure may constrain both individual surgeon expertise and institutional confidence in offering robotic approaches for such cases, especially when open outcomes are well established.

Anatomic and equipment-related considerations further complicate adoption in children. Contemporary robotic platforms are primarily designed for adult anatomy, with instrument size, port spacing, and arm configuration optimized for larger working spaces [33, 34]. Although newer-generation systems have reduced the footprint of robotic arms and can be safely deployed in children when ports are placed at least 6 cm apart [7, 34], challenges remain in smaller patients, particularly those under 15 kg [33, 34]. These factors can limit dexterity, restrict optimal port placement, and increase concern for instrument collision or external arm clashing, all of which may dissuade surgeons from attempting complex reconstructions robotically in younger or smaller children. Economic and system-level constraints also play a central role. Robotic platforms require substantial capital investment, ongoing maintenance costs, and per-case disposable expenses. In many institutions, finite robotic resources are preferentially allocated to high volume procedures with more predictable operative times and shorter learning curves, such as pyeloplasty or ureteral reimplantation [35, 36]. In lower-volume pediatric centers, potential ergonomic advantages, shorter hospital stays, or cosmetic benefits may not sufficiently offset higher upfront costs and longer operative times for complex LUT reconstructions. As a result, many institutions may choose to reserve robotic platforms for selected cases and continue to perform complex bladder reconstructions via open approaches that are familiar, reproducible and efficient. Despite these barriers, robotic platforms remain conceptually well-suited to address several intrinsic challenges of complex pediatric reconstruction. Over the past two decades, iterative refinements in robotic systems have improved visualization, instrument articulation, and ergonomics, which can be particularly advantageous in confined pelvic spaces and in reconstructive procedures requiring precise suturing [37]. Emerging pediatric-specific adaptations, including smaller trocars, optimized port placement strategies, and single-port systems, may further enhance feasibility and safety for complex LUT reconstruction [10, 19, 21]. However, as our findings indicate, feasibility alone has not translated into widespread adoption.

The implications for training and health systems are substantial. Further expansion of complex pediatric robotic reconstruction will likely depend on structured training programs, formal proctoring pathways, and regionalization of complex cases [31, 32, 38]. High-volume centers can serve as hubs for concentrated experience, simulation-based training, and mentorship, shortening the learning curve and facilitating safe dissemination [38]. In parallel, robust cost-effectiveness analyses are needed to evaluate whether robotic approaches confer sufficient long-term clinical and economic benefits to justify investment in complex pediatric reconstruction [36, 39]. Such evidence will be crucial for shaping institutional policies and resource allocation.

An additional strategy to facilitate broader adoption of complex robotic lower urinary tract reconstruction may involve the development of collaborative networks between low- and high-volume centers. In such models, high-volume institutions could provide remote mentorship, case planning support, and structured proctoring for surgeons at lower-volume centers during the early phases of program development. Alternatively, regional referral pathways may allow complex cases to be concentrated at specialized centers, enabling greater procedural experience, maintenance of technical proficiency, and more efficient utilization of robotic resources. Such collaborative approaches may help overcome volume-related barriers while maintaining patient safety and surgical quality.

Although a formal comparative assessment of outcomes was not an objective of this review, the reported functional outcomes of robotic procedures appear broadly comparable to those of established open approaches. For continent catheterizable channels, a systematic review and meta-analysis found comparable rates of stomal continence, complications, reintervention, and stenosis, with a shorter postoperative length of stay in the robotic group [22]. Similarly, a single-center decade-long comparison reported comparable continence rates between open and robotic approaches (91.4% vs. 91.2%), with a substantially shorter length of stay following robotic surgery [20]. A multi-institutional study reported continence in 85% of patients following robotic Mitrofanoff creation, increasing to 92% after ancillary procedures, which is within the range reported in established open Mitrofanoff series [27, 41]. For augmentation cystoplasty, available comparative evidence suggests that continence outcomes after robotic reconstruction are comparable to those achieved with open surgery [42]. Likewise, robotic bladder neck procedures have demonstrated continence outcomes comparable to open approaches, although reported continence rates across procedures and series remain heterogeneous, ranging from approximately 60% to 100% [43]. Nevertheless, these findings should be interpreted cautiously, as the available evidence consists predominantly of small, retrospective, single-center, and non-randomized studies, and direct comparative data—particularly for augmentation cystoplasty and BNR—remain limited.

This study has limitations. Reliance on published case reports and series introduces publication bias and may underrepresent unsuccessful cases, negative experiences, or centers that discontinued robotic programs. Our estimates of global procedural volumes and adoption rates were derived from published epidemiological reports and manual extraction of case counts from the literature and therefore may not fully capture true case numbers or regional variability in practice patterns. Furthermore, comprehensive global data on annual open procedural volumes for APV, augmentation cystoplasty, and BNR were unavailable. Consequently, direct comparisons between open and robotic procedural volumes could not be performed, and the estimated robotic adoption rates should be interpreted as exploratory approximations based on the best available published estimates of total procedural volumes. Additionally, many publications reported cases over multi-year intervals without granular year-by-year data necessitating allocation of cases across years and introducing approximation. Limited patient-level and center-specific data precluded adjustment for surgeon experience, institutional resources, and patient selection criteria, all of which are likely to influence adoption and outcomes. Despite these constraints, this analysis provides, to our knowledge, the most comprehensive synthesis to date of global adoption patterns for complex pediatric robotic LUT reconstruction in the contemporary era. By integrating contemporary data with earlier bibliometric findings, we demonstrate that while overall pediatric robotic volume has increased substantially, proportional adoption for high-complexity bladder and continence procedures has remained low and essentially unchanged. The possibility of patient overlap between publications originating from the same institutions cannot be completely excluded. Because granular patient-level data were unavailable in the published studies, definitive identification and adjustment for duplicate reporting was not possible.

Future work should focus on multicenter prospective registries and comparative studies that directly evaluate robotic versus open approaches for APV, augmentation cystoplasty, and BNR, with standardized reporting of functional outcomes, complications, and quality-of-life measures. In addition, ongoing technological development of pediatric adapted robotic platforms and instrumentation, together with structured training as well as proctoring, may help overcome current barriers to adoption [34, 37, 39]. Ultimately, the responsible expansion of robotics into high-complexity pediatric urologic reconstruction will require coordinated advances in technology, training, and health-system investment, guided by robust patient-centered outcomes.

## Conclusion

Despite a nearly threefold increase in overall pediatric robotic surgical volume between 2016 and 2025, the proportional adoption for high-complexity bladder reconstructions has remained low and essentially unchanged. Robotic appendicovesicostomy, augmentation cystoplasty, and bladder neck reconstruction together account for a small fraction of cases, with estimated annual adoption rarely approaching 10% for any procedure. These findings suggest that contemporary growth in pediatric robotic urology is driven predominantly by low-to-moderate-complexity operations. Responsible expansion into complex lower urinary tract reconstruction will require pediatric-focused technology, structured training and proctoring, and robust multicenter data on outcomes and cost-effectiveness.

## Data Availability

The data underlying this study were extracted from previously published articles included in this systematic review. All data generated or analyzed during this study are included in this published article and its supplementary information files. Further details are available from the corresponding author upon reasonable request.
